# The anti-apoptotic protein survivin can improve the prognostication of meningioma patients

**DOI:** 10.1371/journal.pone.0185217

**Published:** 2017-09-27

**Authors:** Theo L. Winther, Sverre H. Torp

**Affiliations:** 1 Departments of Laboratory Medicine, Children’s and Women’s Health, University of Science and Technology, Trondheim, Norway; 2 Pathology and Medical genetics, St. Olavs Hospital, Trondheim, Norway; George Washington University, UNITED STATES

## Abstract

**Background:**

The 2016 WHO histopathological grading includes a substantial within-variation in recurrence risk, and is thus insufficient to predict prognosis after initial surgery of patients suffering from meningiomas. The aim of this study was to compare the prognostic value of the histopathological grading and the conventional biomarker MIB-1 with expression of the anti-apoptotic protein survivin to see if this biomarker could complement recurrence prediction.

**Methods:**

Using immunohistochemistry, the expression of MIB-1 and survivin were determined as labeling indices (LIs) in tissue micro arrays from 160 human meningiomas. The accuracy of prognostication was assessed with receiver operator characteristics analyses and standard survival analyses.

**Results:**

The expression of survivin was significantly associated with both histopathological grade (P = 0.022) and recurrence status (P = 0.035). A survivin LI of 1% was identified as the optimal cutoff value to predict recurrence (P = 0.003), and was proven as more reliable than the histopathological grading (P = 0.497) and MIB-1 expression (P = 0.091). This result was further strengthened in multivariate analyses where survivin expression was revealed as an independent predictor of recurrence-free survival, while the histopathological grading and MIB-1 expression did not reach significance (P ≥ 0.156).

**Conclusions:**

These findings suggest that incorporation of survivin in the clinical practice might be useful as complement for the histopathological grading and should further be evaluated in independent prospective studies.

## Introduction

Meningiomas are the most commonly reported primary brain tumor and accounts for more than one-third of these tumors [1]. To predict clinical aggressiveness and risk of recurrence the WHO classification of central nervous system tumors distinguishes between three malignancy grades with increasing tumor aggressiveness: grade I (benign), grade II (atypical) and grade III (malignant) meningiomas [2]. However, this histopathological grading scheme includes a considerable within-grade variation in recurrence risk [2, 3]. While a significant portion of the benign tumors behaves clinical aggressive with recurrence shortly after surgery [3, 4], a substantial portion of the atypical meningiomas have an indolent behavior with good prognosis and no recurrence after surgery [3].

MIB-1 labeling index (LI) has shown to correlate with tumor growth and has been suggested as a supplement for the histopathological grading for estimation of recurrence risk in several studies [5]. However, the literature indicates caution in the application of this proliferation marker because of substantial overlap of indices between recurrent and non-recurrent meningiomas and between histopathological grades [5, 6]. In addition, it has been difficult to establish a universal cutoff value with MIB-1 that can translate between different laboratories due to a heterogeneous staining pattern of this biomarker [3, 5].

The most important clinical question regarding meningiomas implies the prediction of recurrence after initial treatment [3]. While surgery is the standard treatment, adjuvant radiation therapy has proven to improve local control, disease-free survival, and overall survival in the more aggressive subsets of meningiomas [7]. While adjuvant radiation therapy has proven beneficial in aggressive tumors with great likelihood of recurrence, this treatment has also been associated with several adverse effects [7], and should therefore be avoided. To determine which patient that would likely benefit from adjuvant therapy, a better recurrence risk stratification of patients is required [8].

Suvivin has been characterized as an inhibitor of apoptosis protein [9, 10], and has also shown to play an important role in regulation of cell mitosis [11]. While this protein is overexpressed in several tumors [12–16], no expression has been observed in normal differentiated tissues [17–19]. Increased expression of survivin has also been associated with cells resistance against different treatments including radiation therapy and chemotherapy.

The expression pattern of survivin has been investigated in several tumors, showing that almost all types of tumors have alternative survivin expression profile compared to normal tissue [20]. The expression of this anti-apoptotic protein has also shown significant correlation with clinical aggressiveness and prognosis in several tumors [12–15]. Regarding breast cancer, Boidot et al showed that survivin expression might induce breast tumor proliferation [21]. A specific genotype of survivin (31G/C) has shown to increase the risk of bladder cancer with 2.6 folds in a hospital-based study from India [22]. The same genotype has also been demonstrated to be more frequent in colorectal patients compared to healthy individuals [23], and a risk factor for gastric cancer [24]. Furthermore, studies have suggested that survivin might contribute to the prediction of susceptibility and pathological development to hepatocellular carcinoma [25]. In addition, a significant association between survivin and advanced tumors stage and lymph node metastasis in pancreatic cancer has been demonstrated [26].

Few studies have investigated the prognostic value of survivin in meningiomas, showing varying results. While the expression of this biomarker has shown significant correlation with histopathological grade in other brain tumors, including gliomas [27–29], such a correlation has not been proved in meningiomas [30, 31]. Only two studies have tried to correlate survivin expression and RFS, demonstrating conflicting results with one study showing a significant association, and one study showing no association [30, 31].

The statistical power has among these studies have also varied substantially, making it difficult to determine any definitive implications that can be adopted into the clinical practice. In addition, most of these studies only investigate the relationship between survivin and recurrence without investigating whether this biomarker could be complement or surrogate for the histopathological grading. Application of this biomarker in the clinical practice as more accurate predictors of tumor recurrence might contribute to more personalized treatment and improve the prognosis of patients suffering from meningiomas relative to what is possible in the current clinical management of these tumors.

The aim of this study was to investigate whether the expression of survivin correlates with histopathological grade and compare the clinical usefulness with the 2016 WHO classification and MIB-1 biomarker as a predictor of meningioma recurrence after initial surgery.

## Materials and methods

### Patient selection and clinical data

Patients selection and collection of clinical data have been described earlier [32]. All patients who underwent meningioma surgery at St. Olavs Hospital, Trondheim University Hospital, in Norway over a 10-year period between January 1, 1991, and December 31, 2000, were retrospectively analyzed. Patients under the age of 18, with non-intracranial meningiomas or who received post-operative radiation therapy immediately after surgery were excluded from the material. Six cases were additionally excluded due to insufficient amount of tumor tissue for immunohistochemical assessments.

Clinical data were collected from the hospital’s medical records. Patients well-being before surgery was assessed according to the guidelines of WHO performance status, and the extent of resection was defined according to Simpson Resection Grade. Recurrence free survival (RFS) was defined as the time from initial operation to the date of radiological evidence of significant tumor growth assessed by neuroradiologists at the hospital. This assessment was based on magnetic resonance imaging or computed tomography when magnetic resonance imaging was contraindicated.

Each meningioma case was reviewed independently by a researcher and a senior neuropathologist and classified according to the 2016 WHO classification of brain tumors [2]. For any discrepancies, cases were reviewed and consensus was reached.

### Laboratory work

Extraction of cores (1 mm diameter) were performed using an Alphelys Tissue Arrayer MiniCore^®^ 3 (AH diagnostics) with the corresponding software TMA Designer2. Three cores were extracted from various histological confirmed representative locations in each tumor to compensate for potential heterogeneity. Whole-slide sections were included when insufficient amount of tumor tissue were available for TMA construction (n = 19).

**Fig 1:**
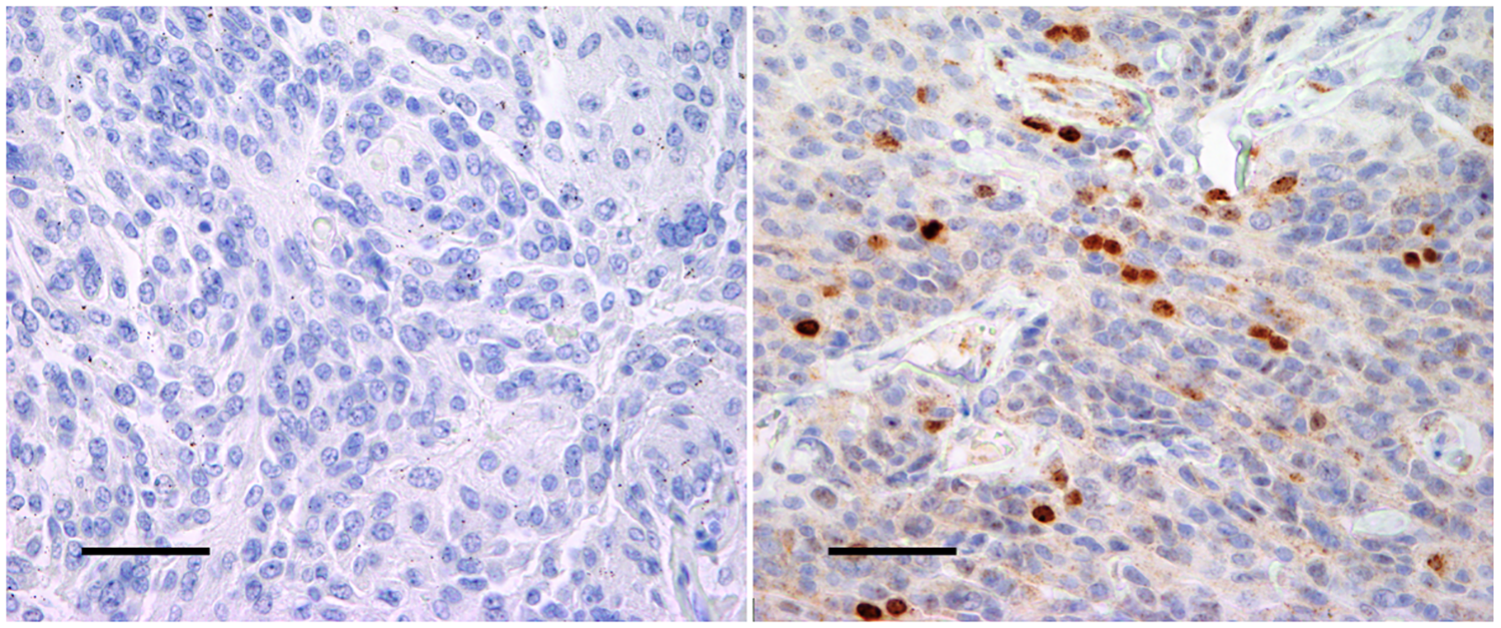
Positive and negative controls. The figures show positive control (A) and negative control (B) from atypical meningiomas (magnitude 400x). The bar on the figures represent 100 μm.

Standard immunohistochemical procedures were applied, using anti-MIB-1 (clone MIB-1, dilution 1:50; Dako Denmark AS, Glostrup, Denmark) and anti-survivin (clone EP288Y, dilution 1:100, Abcam Products, Cambridge, UK) using an automatic Dako Techmate 500. This procedure included a preheating for 1 hour at 60°C and blocking of endogen peroxidase activity with 0.03% H_2_O_2_ for 10 minutes. Incubation was performed, in addition to pre-treating using PT Link Dako. Counterstaining was performed with hematoxylin for all sections. Positive and negative controls were included in each staining (Fig 1). Optimal working dilution was found by titration.

### Assessment of MIB-1 and survivin expression

All meningioma cases were scored with a labeling index (LI) for each antibody based on the percentage of positive immunoreactive nuclei among 1000 tumor nuclei in the area of highest immunoreactivity (hotspots). All assessments were performed by two authors to ensure optimal reproducibility. Both were blinded for clinical data associated with each case during the assessments.

### Statistics

SPSS version 21.0 (SPSS Inc., Chicago, IL) was applied for statistical analyses. The Mann-Whitney U test was used to evaluate the association between the three immunohistochemical markers and histopathological grade and recurrence status. Receiver operator characteristic (ROC) was utilized to determine the optimal cutoff value (based on maximization of the Youden index) in order to discriminate recurrent and non-recurrent meningiomas, and the discriminatory power was tested with the Chi-square test of association. The survival analyses were performed using univariate and multivariate Cox proportional-hazards regression analyses and Kaplan-Meier curves. A P-value equal to or less than 0.05 was considered statistically significant.

### Ethics

This study was approved by the Regional Committee for Medical and Health Research Ethics Central Norway (project number 4.2006.947), and the study protocol adhered to guidelines by Helsinki Convention. Waiver of consent was given by the Regional Ethics Committee because patients were either deceased or severely disabled.

## Results

### Clinical data

A summary of the clinicopathological data according to WHO grade is presented in Table 1. One hundred sixty patients were included for statistical analysis. The median age for all patients was 60 years (range 25–86). One hundred twenty (75.0%) patients were females and forty (25.0%) were males (ratio 3:1). According to the 2016 WHO grading, 100 meningiomas were classified as benign and 60 as atypical. GTR was achieved in 121 (75.6%) patients, while 39 (24.4) patients received STR. The RFS rate for the whole follow-up time was 77.7%. Median follow-up time was 416 weeks (range 0–416).

**Table 1:** Clinical data.

Clinicopatholgical features # (%)	Grade I + II (N = 160)	Grade I (N = 100)	Grade II (N = 60)
Median age (range)	60 (25–86)	58 (27–84)	64 (25–86)
Sex			
Female	120 (75.0)	90 (77.6)	30 (68.2)
Male	40 (25.0)	26 (22.4)	14 (31.8)
Simpson grade			
GTR	121 (75.6)	84 (72.4)	37 (84.1)
STR	39 (24.4)	32 (27.6)	7 (15.9)
WHO performance status			
0–1	134 (83.8)	97 (83.6)	37 (84.1)
2–5	26 (16.2)	19 (16.4)	7 (15.9)
Recurrence			
Yes	30 (18.8)	20 (17.2)	10 (22.7)
No	130 (81.2)	96 (82.8)	34 (77.3)

GTR indicates gross-total resection (Simpson grade I-II) and STR indicates sub-total resection (Simpson grade III-IV).

### Immunohistochemical staining

**Fig 2:**
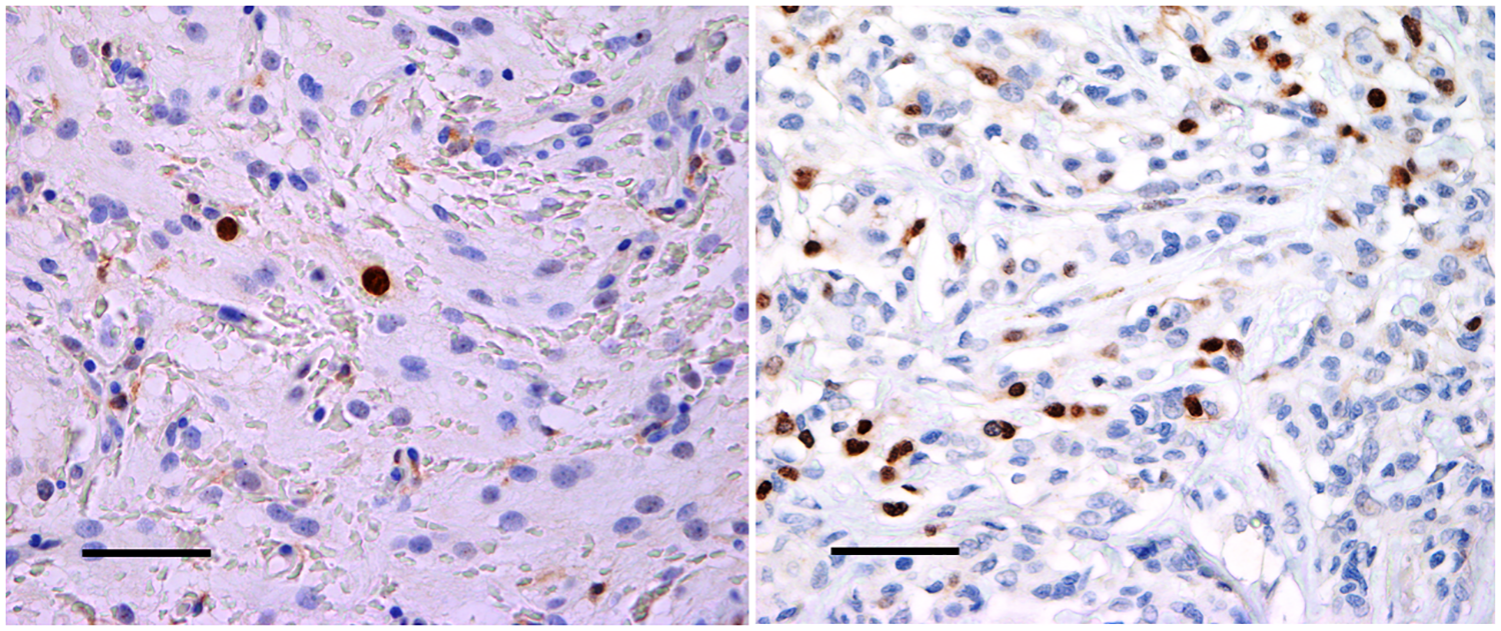
Immunostaining of anti-survivin. Examples of survivin immunostaining in a non-recurrent meningioma (A) and a recurrent meningioma (B) (magnitude 400x). The expression of survivin was significantly higher in recurrent meningiomas compared with non-recurrent meningiomas. The bar on the figures represent 100 μm.

Satisfactory immunohistochemical staining was achieved for both MIB-1 and anti-survivin (Fig 2). Immunoreactivity was confined to the tumor cell nuclei for MIB-1 whereas survivin revealed both nuclear and cytoplasmic reaction. The staining intensity was more homogenous and distinct for survivin compared with MIB-1. MIB-1staining pattern was more heterogeneous with nuclear accentuation.

### Comparisons of indices

Table 2 shows an overview of the LIs related to histopathological grade and recurrence status. Both the expressions of MIB-1 and survivin were higher in grade II meningiomas compared with grade I (P ≤ 0.022). The median LIs of MIB-1 were 0.9 and 1.8 (grade I vs. grade II, P < 0.001), while the median LIs of survivin were 0.5 and 0.7 respectively (P = 0.022).

**Table 2:** Comparisons of labelling indices.

	MIB-1 LI	Survivin LI
A) WHO grade		
Grade I	0.9 (0.0–5.3)	0.5 (0.0–3.2)
Grade II	1.8 (0.4–6.4)	0.7 (0.0–7.6)
P-value	< 0.001*	0.022*
B) Recurrent/non-recurrent		
Non-recurrent	1.2 (0.0–6.4)	0.5 (0.4.3)
Recurrent	1.1 (0.2–6.2)	0.8 (0.0–7.6)
P-value	0.854	0.035*

Differences in proliferation indices between WHO grades (A), and recurrent and non-recurrent meningiomas (B). Mitotic index is defined as the number of mitotic figures per 10 consecutive high power fields, and PI indicates proliferative index defined as the percentage of positive immunoreactive nuclei among 1000 tumor nuclei.

All data are given as median (range).

P-values are calculated by Mann-Whitney U test.

*Significant association, P < 0.05.

**Fig 3:**
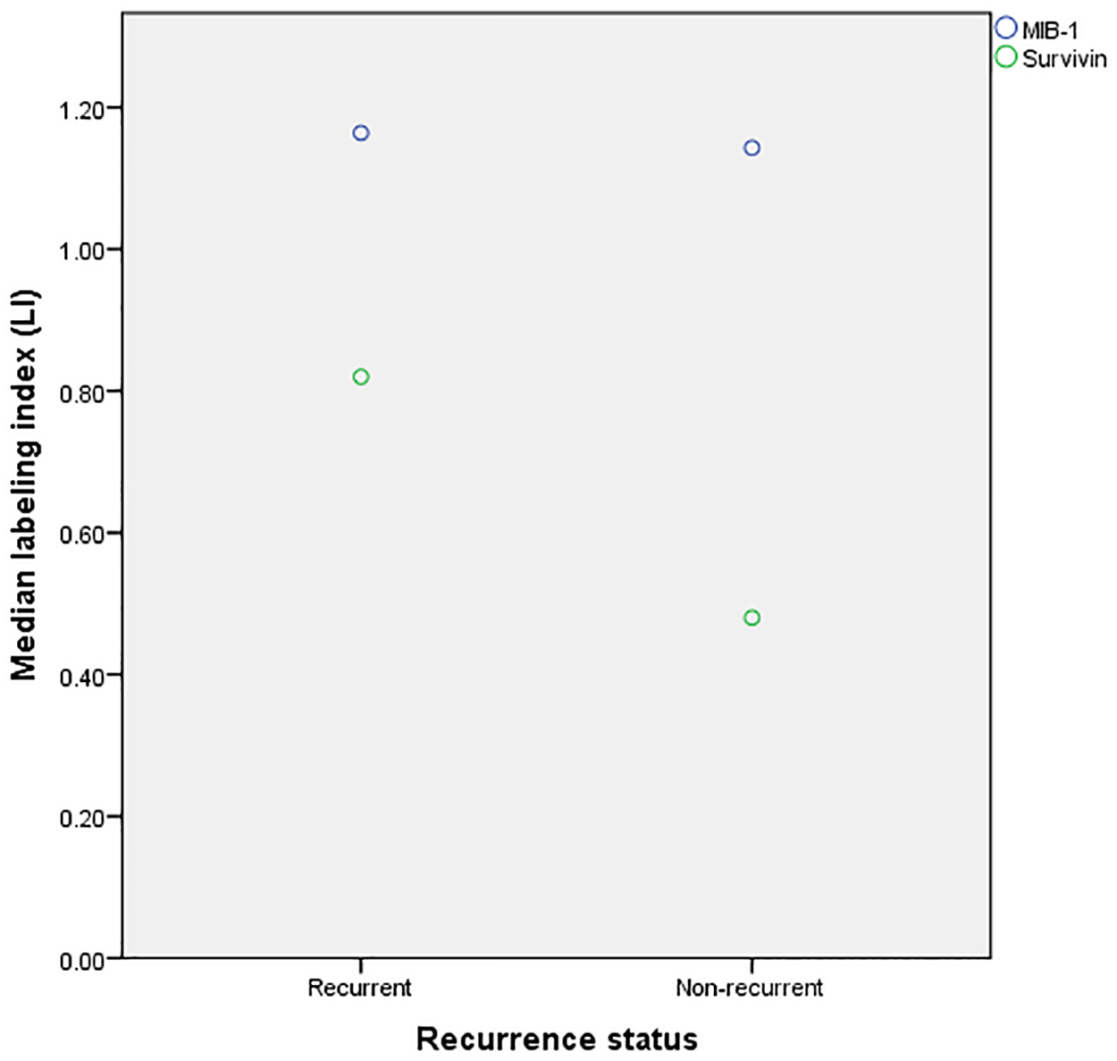
Grouped scatter plot of labeling indices in recurrent vs. non-recurrent meningiomas. Survivin LIs were significantly higher in recurrent meningiomas compared to non-recurrent meningiomas, while no difference was found in MIB-1 LI.

No association was found between MIB-1 expression and recurrence status, with median LI of 1.2 and 1.1 in non-recurrent and recurrent meningiomas, respectively (P = 0.584). The expression of survivin was, however, statistical significantly lower in non-recurrent meningiomas compared with recurrent tumors with median LI’s of 0.5 and 0.8, respectively (P = 0.035) (Fig 3).

### ROC analyses

Based on maximization of Youden index, the optimal cutoff values with respect to recurrence prediction for MIB-1 LI and survivin LI were 3.0% and 1%, respectively (Table 3). Compared with the histopathological grade (grade I vs. grade II) and MIB-1 LI, the expression of survivin was the most accurate predictor of recurrence, revealing highest sensitivity and greatest area under ROC curve (0.64). The cutoff value of survivin LI was also the only significant predictor recurrence (P = 0.003), while histopathological grade and MIB-1 LI did not reach statistical significance (P ≥ 0.091).

**Table 3:** Receiver operator characteristics (ROC) analyses of cutoff values.

	Sensitivity (%)	Specificity (%)	Area under the curve	P-value
WHO Grade	33.3	73.8	0.54	0.497
MIB-1 PI ≥ 3%	26.7	86.9	0.57	0.091
Survivin ≥ 0.990	46.7	80.8	0.64	0.003*

The accuracy of the proliferation assessment methods regarding the distinction between recurrent and non-recurrent meningiomas. Sensitivity, specificity and area under the curve are given by receiver operator characteristics (ROC), and P-values are given by Chi-square test of association.

*Significant association, P < 0.05.

### Survival analyses

**Fig 4:**
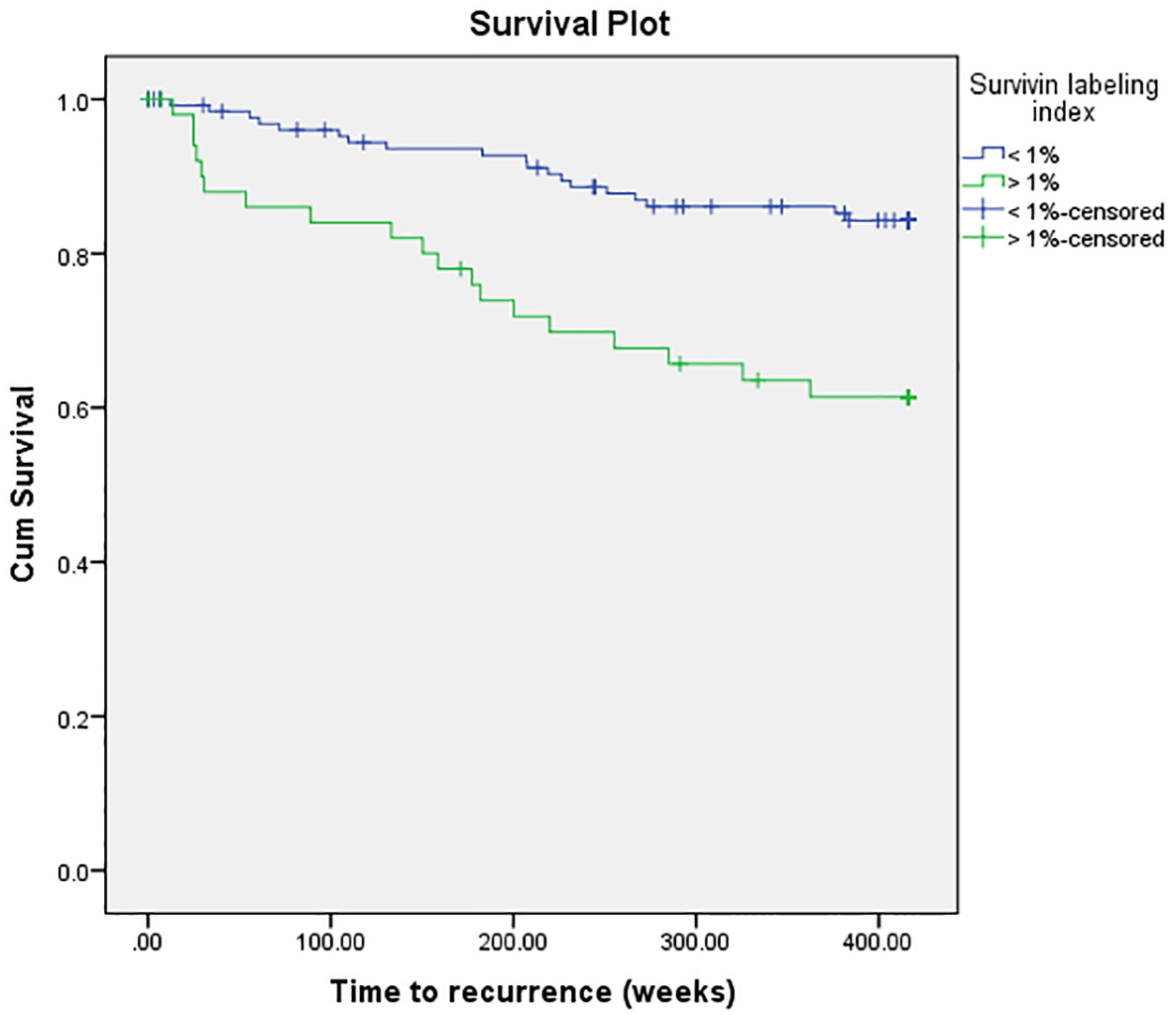
Kaplan-Meier plot of survivin labeling index (LI) < 1% vs. > 1%. Patients with survivin LI > 1% had significant shorter recurrence-free survival compared to patients with survivin LI < 1%.

Survivin expression was revealed as a predictor of RFS in univariate survival analysis (P = 0.002) (Table 4) (Fig 4), while histopathological grade and MIB-1 expression did not reach significance (P ≥ 0.127). Patients with survivin LI ≥ 1% was associated with an increased hazard ratio (HR) of 3.15 compared with patients with survivin expression lower than this cutoff value (P = 0.002).

**Table 4:** Cox hazard univariate and multivariate survival analyses of proliferation assessment methods.

	Hazard ratio (95% CI)	P-value
**Univariate analyses**		
WHO Grade	1.38 (0.64–2.94)	0.410
MIB-1 ≥ 3%	1.88 (0.84–4.22)	0.127
Survivin ≥ 2%	3.15 (1.54–6.45)	0.002*
**Multivariate analyses**		
A) WHO Grade and clinical variables		
Age	1.27 (0.61–2.62)	0.526
Simpson grade	5.31 (2.52–11.19)	< 0.001*
WHO performance status	1.07 (0.43–2.68)	0.879
WHO Grade	1.76 (0.81–3.85)	0.156
B) MIB-1 and clinical variables		
Age	1.00 (0.97–1.02)	0.739
Simpson grade	4.79 (2.31–9.93)	< 0.001*
WHO performance status	1.16 (0.47–2.85)	0.750
MIB-1 ≥ 3%	1.80 (0.80–4.05)	0.158
C) Survivin and clinical variables		
Age	1.37 (0.66–2.84)	0.406
Simpson grade	4.52 (2.16–9.47)	< 0.001*
WHO performance status	1.37 (0.55–3.40)	0.503
Survivin	2.94 (1.42–6.12)	0.004*

Association between recurrence-free survival and the proliferation assessment methods. The multivariate analyses are adjusted for clinical relevant variables. The date of surgery was used as reference for the calculation of recurrence-free survival.

*Significant association, P < 0.05.

In multivariate survival analyses histopathological grade and the LI’s were adjusted for clinically known and relevant variables (age, Simpson Grade and WHO performance status), revealing survivin expression as the only independent predictor of RFS (HR = 2.94, P = 0.004). MIB-1 LI and histopathological grade remained as insignificant predictors of RFS (P ≥ 0.156).

## Discussion

In this present study, we evaluated the prognostic value of nuclear survivin expression and compared the reliability of this biomarker with histopathological grade and MIB-1. We have showed that recurrent tumors have increased expression of survivin compared to non-recurrent tumors, and demonstrated a more accurate prediction of recurrence using this biomarker. We therefore suggest that survivin might be superior to histopathological grade and MIB-1 in predicting recurrence and RFS after initial resection of meningiomas.

Immunohistochemistry is widely used in meningiomas, both for diagnostic and prognostic purposes. The majority of meningiomas stains positively for EMA, vimentin and somatostatin receptor 2A, but none of these are specific for this tumor. Studies have demonstrated that vimentin filaments are anchored to desmosomal plaques. This combination of vimentin and desmosomal plaque proteins, however, is highly specific for arachnoidal cells [33]. Some meningiomas express S-100 protein, positivity for this protein is most commonly found in the fibrous subtype. As opposed to most carcinomas, staining for cytokeratin is rarely positive, and the same applies to GFAP.

Several biomarkers have proven to be associated with prognosis and recurrence rate [34–41]. Some of the most interesting markers include the progesterone receptor, MIB-1 and PHH3. Expression of progesterone receptor is inversely correlated with histopathological grade and prognosis, however, this correlation is not optimal [42]. PHH3 is considered as a mitosis specific marker which facilitates mitotic count. Even though studies have showed that this biomarker can compensate for several weaknesses associated with the WHO classification, the association with prognosis remains imperfect [43]. Other interesting immunohistochemical markers with potential include cathepsin D, E-cadherin, claudin and platelet-derived growth factor receptor beta [44]. However, most of these markers lack specificity. The search for novel prognostic markers should therefore continue, and in this setting survivin has shown potential.

The observed association between survivin expression and histopathological grade is somewhat in conflict with the previous literature. Investigating the survivin expression in 86 atypical and malignant meningiomas, Vranic et al. could not prove any association with histopathological grade [30]. This observation is consistent with the findings of Pfister et al. who used a method based on PCR [31]. Kaoyaselecah et al. were able to prove a significant difference in survivin values between benign and malign meningiomas, however, no difference in values was found between benign and atypical meningiomas or between atypical and malign meningiomas [45]. These insignificant observations might be explained by the small number of patients included in these studies, with ≤ 100 patients in total and only ≤ 10 patients classified into at least one of the histopathological groups. In contrast, we included 160 patients in total and classified them into two histopathological groups with 100 and 60 patients (with benign and atypical meningiomas, respectively). Since the difference in survivin expression is quite small (median values of 0.5 and 0.7 in benign and atypical meningiomas, respectively) a large number of patients is required to demonstrate a statistical significant difference. Furthermore, while the past studies in the literature used one of the previous WHO classification guidelines, we reviewed all our cases and classified them according to the 2016 WHO classification guidelines [2]. This is the first study to demonstrate an association between survivin expression and histopathological grade in meningiomas, however, similar association has previously been described in studies investigating survivin expression in other brain tumors [27–29].

Survivin expression also showed a significant association with recurrence status, revealing overexpression in recurrent tumors compared with non-recurrent meningiomas. We are only aware of two previous studies that have been looking for an association between survivin expression and recurrence status, with one showing a significant association [45], while one did not [46]. An explanation for the disparate results might be the variation in study inclusion criteria. Das et al. included only surgically gross total resected benign meningiomas in their study, and thus only 12 of their patients experienced recurrence [46]. Excluding patients with smaller extent of resection might therefore have led to exclusion of a number of recurrent tumors. In addition, 17 of their patient underwent preoperative embolization which might have led to better tumor control and thus lower recurrence rate in these tumors, which might also have affected the results. In contrast, we included all surgically resected patients independent of extent of resection and not a single patient in our study received preoperative embolization or any adjuvant postoperative treatment.

We also extended our study to investigate whether survivin expression is associated with RFS and demonstrated that the survivin levels in meningiomas was an independent predictor of RFS. Previous literature has been divided in this area, with one study demonstrating survivin expression as predictive of recurrence free survival (RFS) [31], while one study showing no such association [30]. However, this previous negative study measured survivin expression as a score from 1 to 3 based on the approximate percentage of stained cells. Only dividing tumors into three groups, this method causes a wide within-group heterogeneity with regard to anti-apoptotic potential of meningiomas. Hence, a more accurate method is required to measure this potential. Hence, we used a LI—the number of positive labeled tumor nuclei among 1000 tumor nuclei—as a more accurate measurement of survivin expression in tumors, and found a significant correlation between survivin expression and RFS.

MIB-1 is the currently used prognostic biomarker in clinical practice in human meningiomas. However, several limitations exist in the application of this biomarker related to a substantial overlap of indices between histopathological grades and between recurrent and non-recurrent tumors [5, 6]. Furthermore, no universal cutoff value has been established in the literature with respect to RFS prediction because of a wide range of different values suggested in different studies, ranging from 1% to 10% [5]. Previous literature is also ambiguous on whether increased MIB-1 expression is associated with recurrence risk, with some studies demonstrating an association [47, 48], while others do not [5, 6]. In our survival analyses, no significant association between MIB-1 expression and RFS was found. We therefore suggest some caution using MIB-1 as a prognostic marker in clinical practice [6].

While prior studies on survivin expression have been limited to evaluate the possible associations between survivin expression and recurrence status or RFS, we extended our analysis to compare the prognostic value of this biomarker with histopathological grade and MIB-1. Based on ROC analysis, we proved survivin expression as the most accurate predictor of recurrence in meningiomas, revealing the greatest area under curve (AUC), highest sensitivity and the only significant association tested by Chi-Square test of association. This resulted in a correct identification of 14 recurrent tumors, while histopathological grade and MIB-1 were only able to correctly identify 10 and 8 recurrent tumors, respectively. Furthermore, survivin expression was additionally the only significant predictor of RFS in survival analyses and achieved the highest hazard ratio. We therefore suggest survivin expression as a more accurate predictor of prognosis in patients after initial meningioma surgery compared with histopathological grade and MIB-1 proliferation marker and thus more useful in a clinical setting.

Patients with survivin expression exceeding approximately 1% might benefit from a more aggressive treatment approach, such as adjuvant radiation therapy with the possibility of re-resection, in addition to more frequent radiological follow-up. In contrast, patients with low expression of survivin below the proposed cutoff value periodically radiological follow-up is likely sufficient, with re-resection and/or radiation therapy in case of recurrence. However, other risk factors must also be taken into consideration regarding treatment decision-making, and the risk of surgery and adjuvant therapy must always be weighed against the possible benefits. In addition, as with every immunohistochemical test, labeling indices may differ between laboratories. Hence, extrapolating other laboratories’ values is problematic. It is therefore recommended that each laboratory establish its own practice. Such a policy is also valuable as a quality assurance at a pathology department.

Our study has limitations inherent to the nature of retrospective studies and immunohistochemical analyses at a single institution. Furthermore, the assessment of proliferation on TMA might be complicated by a heterogeneous expression pattern of immunohistochemical markers. However, we tried to compensate for this pitfall by extracting cores from three different histologically confirmed representative areas. Moreover, because construction of TMA cylinders is a resource intensive process, this method is not as relevant in daily clinical practice as it is for research purposes. In addition, recurrence which was used as the end point parameter does not always equal worsening of symptoms or poor outcome, and the impact on the patients’ quality of life will vary. As with all immunohistochemical analyses, this study requires investigation in independent cohorts for verification and optimization of cutoff values and clinical usefulness.

In conclusion, we found higher expressions of survivin in recurrent meningiomas compared with non-recurrent ones. Survivin expression with a cutoff of 1% was identified as the most accurate predictor of tumor recurrence compared with the 2016 WHO histopathological grading and MIB-1 proliferation marker. Furthermore, survivin was demonstrated as the only significant predictor of RFS when controlled for other clinical factors. These findings suggest that incorporation of survivin in the clinical practice might be useful and should further be evaluated in independent prospective studies. Survivin might also be a pharmacological target for these patients, and can lead to medical treatment of meningiomas, which currently does not exist.
